# Environmental Justice: The Tuskegee Legacy Project

**DOI:** 10.1289/ehp.115-a130a

**Published:** 2007-03

**Authors:** Luz Claudio

Medical research studies often do not include ethnic and racial minorities as study participants in numbers that are representative of their populations. A study published in the November 2006 issue of the *Journal of Health Care for the Poor and Underserved* aimed to determine whether the paucity of minorities included in research could be explained by differences in willingness and misgivings related to participation in health research.

The study, funded by the National Institute of Dental and Craniofacial Surgery, was conducted by a research team within the Tuskegee Legacy Project (TLP), which was inspired by a 1994 bioethics conference at the University of Virginia. The research team was created to assess how the infamous Tuskegee Syphilis Study affected the attitudes of black Americans toward health research. From 1932 to 1972, 399 black men with syphilis were studied to observe the effects of untreated syphilis, even though effective treatment was already available. This unethical study has often been used to explain the assumption that blacks may be more prone than whites to distrust research and refuse to participate.

To test this assumption, the research team developed the TLP Questionnaire, which contained two scales: the Likelihood of Participation Scale and the Guinea Pig Fear Factor Scale, which measured self-reported general willingness to participate in research and fear of participating in research, respectively. The TLP Questionnaire was taken by more than 1,000 black, white, and Hispanic residents of four U.S. cities (two in Alabama including Tuskegee, one in Texas, and one in Connecticut).

Significantly, the results showed that only about 30% of all people surveyed expressed a willingness to participate in research studies. Blacks were 1.8 times as likely as whites to fear participating in biomedical research. Still, they were equally as willing to participate in research as whites. “Given the history of the treatment of African-Americans in our country, it makes sense that blacks would have a heightened awareness of potential dangers,” says lead author Ralph V. Katz, chairman of the Department of Epidemiology and Health Promotion at the New York University College of Dentistry.

These findings are consistent with the few other studies in which racial/ethnic differences in research participation have been assessed. They also are particularly important in studies addressing health disparities and those that aim to study environmental justice issues in minority populations. “African Americans come from varied experiences in the health care system. As such, there is no monolithic response to health-seeking behaviors, including participation in health research,” says Ruth Browne, CEO of the Arthur Ashe Institute for Urban Health in Brooklyn, New York. “This really points to the importance of culturally appropriate outreach efforts.”

